# Supplementary material for: Metagenomic Strain-Typing Combined with Isolate Sequencing Provides Increased Resolution of the Genetic Diversity of Campylobacter jejuni Carriage in Wild Birds
Source: Microorganisms. 2023 Jan 3;11(1):121. doi: 10.3390/microorganisms11010121 (PMC9860660; doi:10.3390/microorganisms11010121)
Supplement: Supplementary file 1 [file microorganisms-11-00121-s001.zip › Supplementary_Figure_S1.pdf]

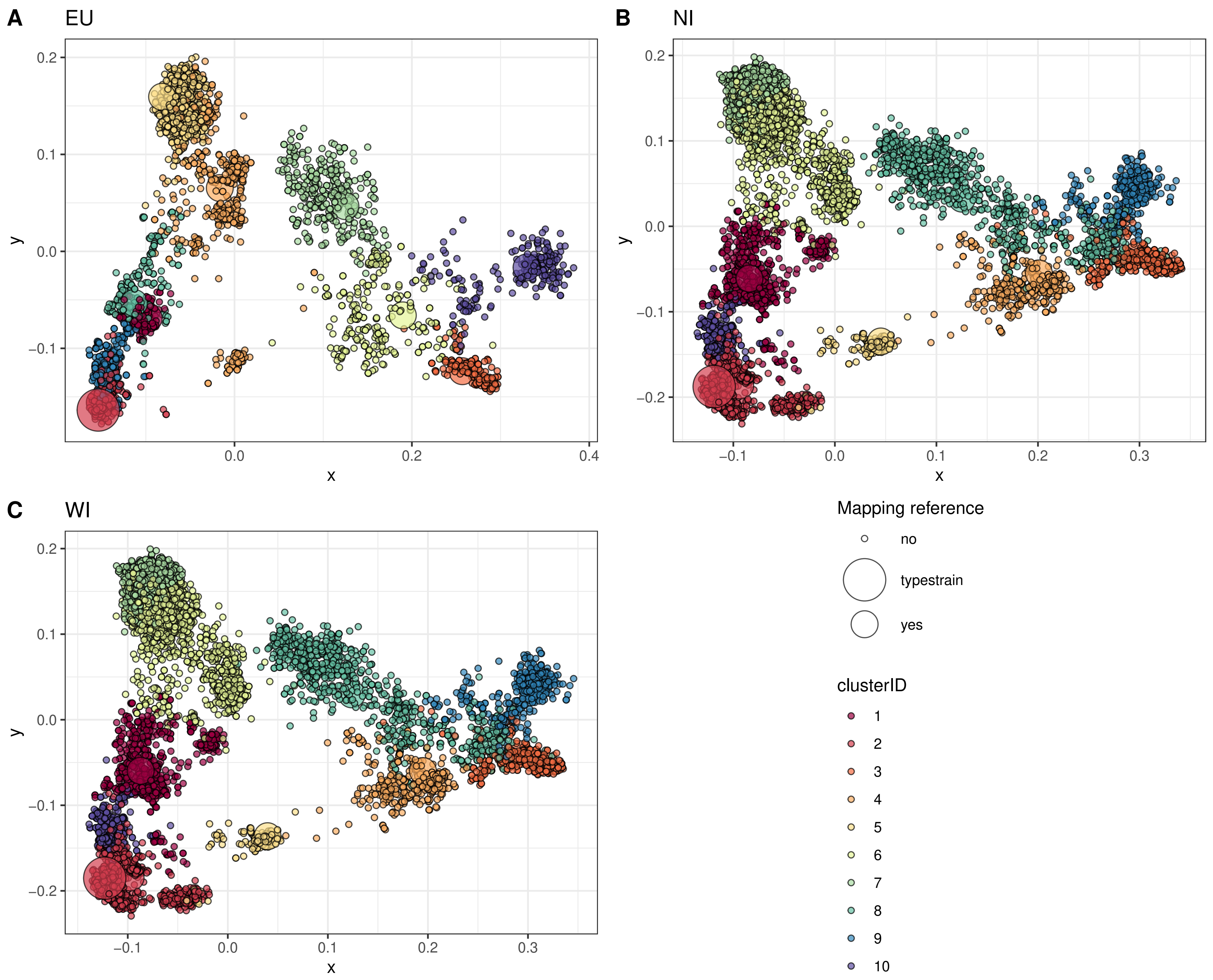

Supplementary Figure S1: PcoA plots of sourmash distances with selected clusters (color) and mapping-representatives highlighted (point size), for the three databases: Including inhouse isolates and only European reference genomes (A), excluding inhouse isolates (B), and including inhouse isolates (C)
