# Supplementary material for: Metagenomic Strain-Typing Combined with Isolate Sequencing Provides Increased Resolution of the Genetic Diversity of Campylobacter jejuni Carriage in Wild Birds
Source: Microorganisms. 2023 Jan 3;11(1):121. doi: 10.3390/microorganisms11010121 (PMC9860660; doi:10.3390/microorganisms11010121)
Supplement: Supplementary file 1 [file microorganisms-11-00121-s001.zip › Supplementary_Figure_S2.pdf]

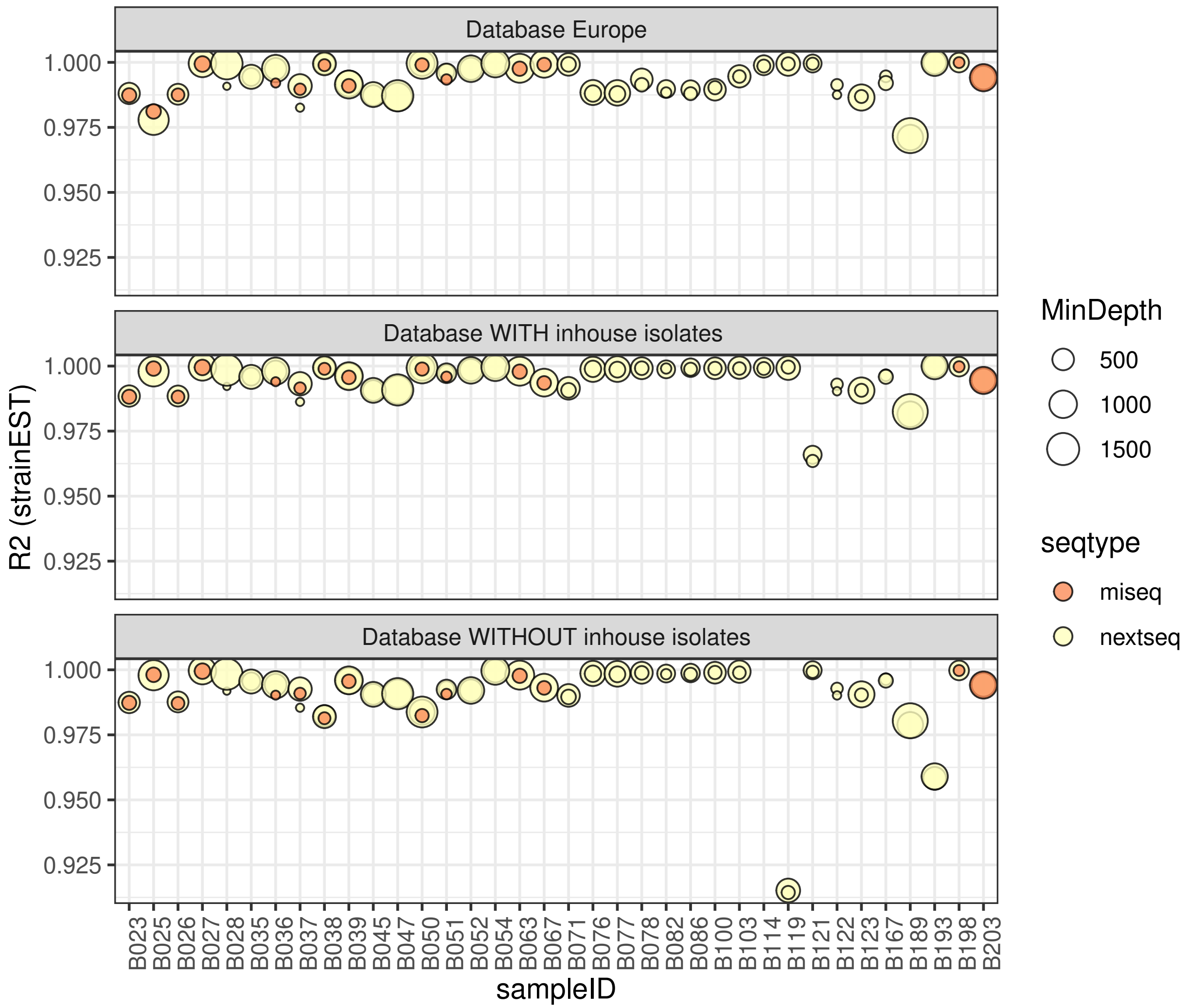

Supplementary Figure S2: Overview of re-sequenced samples for the three databases (EU, WI, and NI) from top to bottom, showing R2-values from StrainEST and MinDepth (point size). Point color indicates the sequencing platform.
