# Supplementary material for: Metagenomic Strain-Typing Combined with Isolate Sequencing Provides Increased Resolution of the Genetic Diversity of Campylobacter jejuni Carriage in Wild Birds
Source: Microorganisms. 2023 Jan 3;11(1):121. doi: 10.3390/microorganisms11010121 (PMC9860660; doi:10.3390/microorganisms11010121)
Supplement: Supplementary file 1 [file microorganisms-11-00121-s001.zip › Supplementary_Figure_S3.pdf]

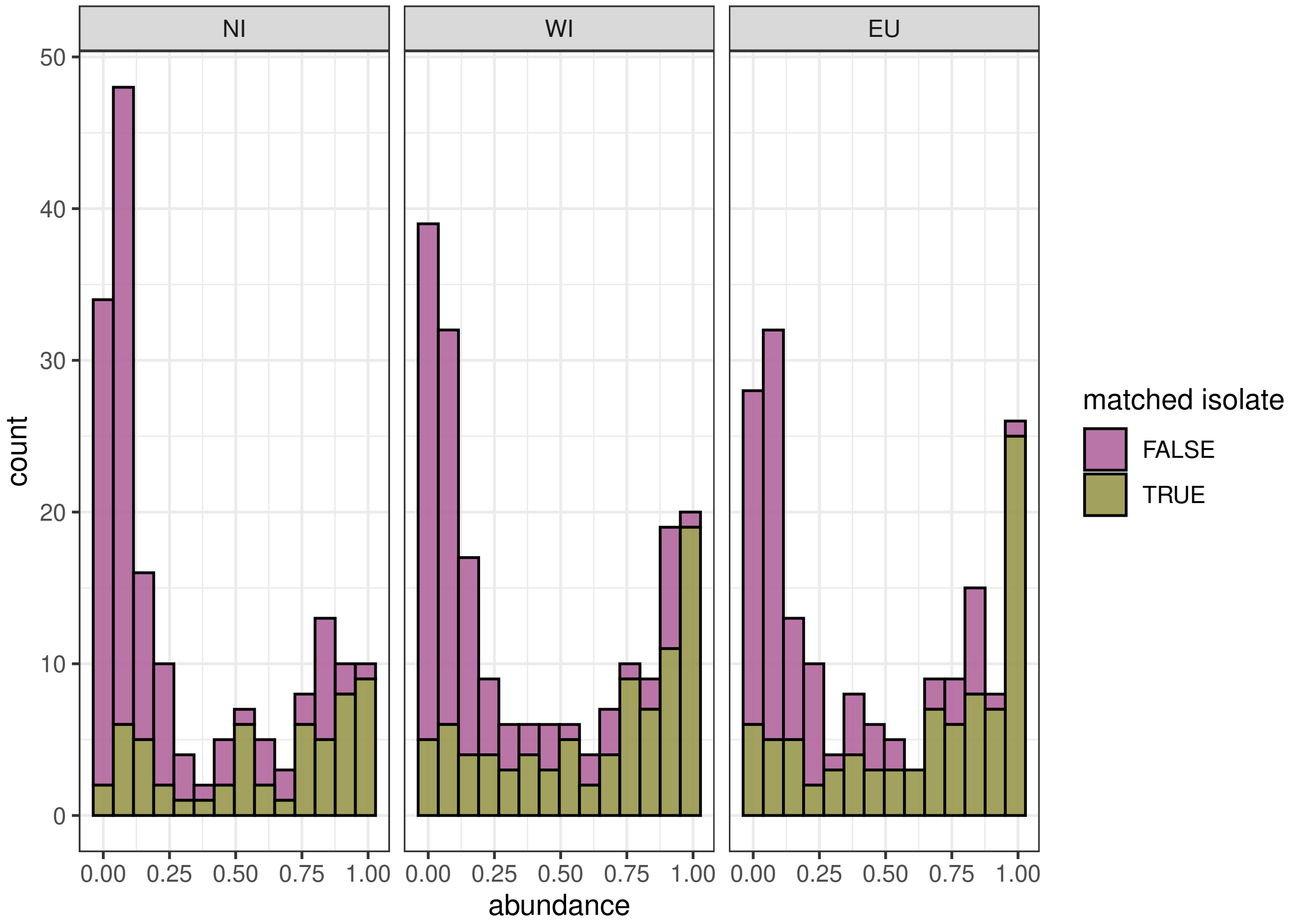

Supplementary Figure S3: Abundance histograms of predicted strain profiles with and without matching isolates. Panels are separated according to databases (NI: without isolates from this study, WI: including isolates from this study, EU: including isolates from this study and only reference genomes from Europe)
